# Gene Expression Profile and Co-Expression Network of Pearl Gentian Grouper under Cold Stress by Integrating Illumina and PacBio Sequences

**DOI:** 10.3390/ani11061745

**Published:** 2021-06-11

**Authors:** Ben-Ben Miao, Su-Fang Niu, Ren-Xie Wu, Zhen-Bang Liang, Bao-Gui Tang, Yun Zhai, Xue-Qi Xu

**Affiliations:** 1 College of Fisheries, Guangdong Ocean University, Zhanjiang 524088, China; benben.miao@outlook.com (B.-B.M.); wolf0487@126.com (S.-F.N.); liangzhenbang0403@163.com (Z.-B.L.); tangbg@gdou.edu.cn (B.-G.T.); zhaiyun1102@163.com (Y.Z.); 15992559950@163.com (X.-Q.X.); 2 Southern Marine Science and Engineering Guangdong Laboratory, Zhanjiang 524025, China

**Keywords:** PPI network, SMRT-Seq, RNA-Seq, WGCNA, STEM analysis

## Abstract

**Simple Summary:**

In this study, we investigated the liver transcriptomic responses of pearl gentian grouper towards cold stress. Some cold-related key genes and biological pathways were screened, of which energy-related metabolic pathways and genes had higher expression levels under cold stress. This suggested that energy homeostasis plays a crucial role in the physiological adjustments of pearl gentian grouper when exposed to the cold stress environment. Our results will expedite the understanding of different fishes adaptive mechanisms to profound environmental temperature changes and provide insights into the molecular breeding of cold-tolerant pearl gentian grouper varieties.

**Abstract:**

Pearl gentian grouper (*Epinephelus fuscoguttatus* ♀ × *Epinephelus lanceolatus* ♂) is a fish of high commercial value in the aquaculture industry in Asia. However, this hybrid fish is not cold-tolerant, and its molecular regulation mechanism underlying cold stress remains largely elusive. This study thus investigated the liver transcriptomic responses of pearl gentian grouper by comparing the gene expression of cold stress groups (20, 15, 12, and 12 °C for 6 h) with that of control group (25 °C) using PacBio SMRT-Seq and Illumina RNA-Seq technologies. In SMRT-Seq analysis, a total of 11,033 full-length transcripts were generated and used as reference sequences for further RNA-Seq analysis. In RNA-Seq analysis, 3271 differentially expressed genes (DEGs), two low-temperature specific modules (tan and blue modules), and two significantly expressed gene sets (profiles 0 and 19) were screened by differential expression analysis, weighted gene co-expression networks analysis (WGCNA), and short time-series expression miner (STEM), respectively. The intersection of the above analyses further revealed some key genes, such as PCK, ALDOB, FBP, G6pC, CPT1A, PPARα, SOCS3, PPP1CC, CYP2J, HMGCR, CDKN1B, and GADD45Bc. These genes were significantly enriched in carbohydrate metabolism, lipid metabolism, signal transduction, and endocrine system pathways. All these pathways were linked to biological functions relevant to cold adaptation, such as energy metabolism, stress-induced cell membrane changes, and transduction of stress signals. Taken together, our study explores an overall and complex regulation network of the functional genes in the liver of pearl gentian grouper, which could benefit the species in preventing damage caused by cold stress.

## 1. Introduction

A change in water temperature could affect homeostasis and physiological functions of aquatic animals. The most directly influenced physiological functions include behavior, survival, growth, development, and reproduction [1,2]. Fish adapt variably to low-temperatures in their migratory environments through different metabolisms, biochemical reactions, and physiological adjustment processes involving numerous genes and biological pathways [1].

Transcriptomics is an effective method envisioned as a valuable tool for identifying key genes and biological pathways of fishes that respond to low environmental temperature. Numerous studies have characterized the transcriptional changes in various fishes as they respond to cold stress and found they exhibited specific physiological responses to their acclimation during low-temperature stress [3,4,5]. For instance, several main pathways, including carbohydrate metabolism, lipid metabolism, immune system, and signal transduction pathways, were significantly enriched in many fishes [6,7,8,9,10]. In contrast, *Sparus aurata* and *Danio rerio* have been resistant to the adverse effects of cold stress through different functional regulations like antioxidant responses, blood glucose regulation, and membrane fluidity [5,11]. Previously performed transcriptomic studies have enriched our understanding of the transcriptional regulations of fish adaptation to low-temperature environments.

However, most previous reports have demonstrated that only short and incomplete transcripts could be obtained from Illumina RNA-Seq, which inevitably limits the complete annotation for species with incomplete reference genomes. In contrast, the single-molecule real-time sequencing (SMRT-Seq) of the Pacific Biosciences (PacBio) application can effectively avoid errors caused by short sequence assembly and variable splicing [12]. Besides, it can obtain up to 20 kbp of high-quality, full-length transcripts and accurately annotate this information [13], which is very beneficial during downstream transcriptomics analyses. Consequently, the developed PacBio SMRT-Seq technique has been effectively applied to reveal transcript diversity and splice isoforms of multiple species and study genetic variation in different species, including *Homo sapiens* [14], *Sus scrofa* [15], and *Manis javanica* [16]. Therefore, the analytical strategy of integrating PacBio SMRT-Seq and Illumina RNA-Seq techniques is necessary to generate comprehensive transcriptome information of species without relying on a genome reference. Moreover, this will comprehensively and accurately reveal the molecular mechanisms underlying the transient response to cold stress by various fish species.

Pearl gentian grouper (*Epinephelus fuscoguttatus* ♀ × *Epinephelus lanceolatus* ♂) is an F_1_ hybrid fish. Compared to its parental species, it has faster growth, stronger disease resistance, and better tolerance to captive conditions [17,18]. Given these biological advantages, the pearl gentian grouper is considered highly suitable for large-scale aquaculture along the coast of Asia [19]. Under captive conditions, its optimum living temperature range is 25–35 °C. When the water temperature drops, the fish would reduce feeding at 24 °C, stop feed at 13 °C, and start dying at 11 °C [20]. Therefore, the current area under aquaculture for this fish species is mainly limited to the coastal waters of southern China (e.g., Fujian, Guangdong, Guangxi, Hainan province) [21]. However, this region is not conducive for further expansion of aquaculture area and scale. Notably, during a recent winter season in southern China, cold fronts occurred frequently [11,22], resulting in a sharp water temperature decline in these coastal aquaculture areas, which attained a minimum of 5 °C (Guangdong province) [21]. Subsequently, the feeding, growth, and survival rates of these coastal cultured fishes were significantly decreased [23], causing substantial economic losses in their aquaculture [11]. The above facts would inevitably influence the production benefit of culturing pearl gentian grouper, affecting its aquaculture industry output. Therefore, whether it can adapt to low temperature directly affects the survival, feeding, and growth of Pearl gentian group. Nevertheless, previous studies mainly focused on feed nutrition and pathology [24,25,26,27] and did not involve research on gene expression related to low temperature. Besides, the molecular regulation mechanism underlying cold stress in pearl gentian grouper species remains largely unidentified. Thus, there is a need to analyze the transcriptomic responses towards cold stress of pearl gentian grouper to provide an essential basis for studying cold adaptability and genetic improvement for breeding this species.

This study investigated the transcriptomic responses of pearl gentian grouper towards cold stress using Illumina RNA-seq and PacBio SMRT-Seq technologies. Taken together, this study aimed at identifying some critical genes and biological pathways closely related to cold stress using an integrated analysis of RNA-seq, short time-series expression miner (STEM) program analysis, and weighted gene co-expression network analysis (WGCNA). On this basis, we further explored the molecular regulatory mechanisms of pearl gentian grouper under cold stress conditions. These findings will expedite our understanding of fish adaptive mechanisms to profound environmental temperature changes and provide insights into the molecular breeding of cold-tolerant varieties.

## 2. Materials and Methods

### 2.1. Cold Stress Experiment and RNA Extraction

A total of 200 healthy juveniles of pearl gentian grouper were purchased from Zhanjiang Hist Aquatic Technology Co., Ltd. in September 2019, kept alive in oxygen bags, and transported to the experimental base. The average standard lengths and weights of the above fishes were 10.57 ± 1.63 cm and 36.94 ± 11.46 g, respectively. The parents of these fishes came from the pearl gentian grouper breeding population in Hainan, which is also one of the main sources of the species along the coast of southern China. Before starting the experiment, fishes were approximately aliquoted into three plastic tanks (1.0 m ×1.0 m × 1.5 m) and were acclimated to a laboratory condition for two weeks. The seawater quality for acclimation is as follows: temperature (25.0 ± 1 °C), salinity (24 ± 1‰), and dissolved oxygen (6.0 ± 0.3 mg/L). During the acclimation period, fishes were fed with commercial pellet feeds twice daily, and approximately 30~40% of holding water was renewed once daily to ascertain a proper growth environment. 

Before commencing the cold stress experiment, the fishes in three parallel experimental tanks were fasted for 24 h. After the experiment started, the water temperature in three parallel experimental tanks was gradually decreased from 25 to 12 °C at a constant rate of 1 °C/h, and then kept at 12 °C for 6 h using a cooling-water machine (CW0500). Three fishes were randomly sampled from each of the three tanks at temperature points of 25, 20, 15, 12, and 12 °C kept for 6 h. Thus, a total of 45 samples were obtained from the control group (CT) and four treatment groups respectively named LT20, LT15, LT12, and LT12-6 groups. Subsequently, liver tissues were collected and immediately frozen in liquid nitrogen.

Total RNA was isolated from each liver tissue of 45 samples by TRIzol reagent kit (Invitrogen, Carlsbad, CA, USA) following the instruction from the manufacturer. The residual genomic DNA fragments were removed from the extracted total RNA using DNase I (Promega, Madison, WI, USA). Lastly, RNA concentration, purification, and RNA integrity number (RIN) values were detected using Qubit 3.0 Fluorometer (Invitrogen, NY, USA), Nanodrop 2000 Spectrophotometer (Thermo Fisher Scientific, Waltham, MA, USA), and Agilent 2100 Bioanalyzer (Agilent Technologies, Palo Alto, CA, USA), respectively. Finally, 15 better quality RNA samples from 45 samples (taking one sample per parallel tank of all groups) were selected for constructing sequencing libraries with different strategies of PacBio SMRT-Seq and Illumina RNA-seq. 

### 2.2. PacBio SMRT-Seq and Analysis

#### 2.2.1. Library Construction and Sequencing

The RNAs of 15 samples were mixed equally (pooled RNAs) for PacBio SMRT-Seq library construction and subjected to full-length transcriptome sequencing that was carried out at Wuhan Frasergen Gene Information Co., Ltd. (Hubei, China). The full-length cDNA of mRNA was synthesized using SMARTer cDNA synthesis kit. The obtained full-length cDNA was amplified through PCR, and the product purified using PB magnetic beads and the libraries quantified using Qubit 3.0 Fluorometer. Subsequently, the full-length cDNA was end-repaired and the SMRT dumbbell adapter was connected for sequencing. Lastly, the size of the resulting library was validated using an Agilent 2100 Bioanalyzer. After its size and purity were verified and confirmed to meet expectations, the library was sequenced using the PacBio Sequel System.

#### 2.2.2. Transcripts Assembly and Annotation of PacBio SMRT-Seq

The raw read data of PacBio SMRT-Seq were preprocessed using SMRTlink v8.0.0 pipeline [28] from the Pacific Biosciences to remove any residual sequencing adapters, and low-quality sequences full-length transcripts were obtained through the Iso-Seq analysis process. The polymerase reads of SMRT-Seq were split into multiple reads (called subreads), and the circular consensus sequence (CCS) reads were extracted from the subreads BAM file format. The CCS reads were classified into full-length non-chimera (FLNC), full-length chimeras (FLC), non-full-length (NFL), poly-A sequence, and short reads based on the cDNA primers. Notably, all reads shorter than 50 bp were removed from the sequence data according to the parameter setting. FLNC reads with similar motifs were clustered using the SMRTlink Iterative Clustering software tool for Error Correction (ICE) under the following conditions: the length difference at the 5′ end <100 bp, the length difference at the 3′ end <30 bp, or the gap length within the sequence <10 bp. Then, the cluster consensus isoforms were further polished using an Arrow tool in the SMRTlink software. Next, the polished transcripts were corrected using the consensus sequence of Illumina sequencing clean reads by Lordec v0.9 [29]. Finally, the redundant sequences were removed from corrected polished transcripts using the CD-HIT v4.8.1 software [30] with 0.99 clustering threshold, and the final full-length transcripts were generated.

The full-length transcripts pathways were annotated based on several databases, including NCBI Non-redundant Protein (NR), Kyoto Encyclopedia of Genes and Genomes (KEGG), Gene Ontology (GO), Universal Protein Resource (UniProt-SwissProt), and Eukaryotic Ortholog Groups (KOG) using DIAMOND program [31].

### 2.3. Illumina RNA-Seq and Analysis

#### 2.3.1. Library Construction and Sequencing

After total RNA extraction and quality check, the mRNA was enriched separately from each of the 15 RNA samples using the Oligo (dT) magnetic beads and then fragmented into short fragments using a fragmentation buffer. The cleaved RNA fragments were reverse transcribed into the first-strand cDNA, and the blunt-ended double-stranded cDNA was purified using AMPure XP beads. Subsequently, the purified cDNAs were end-repaired, poly-A were added, Read1 and Read2 sequencing primers were connected, PE adapters were ligated, and Index sequence was inserted between the adapter and the sequencing primer. Suitable fragments (about 200 bp) were selected using AMPure XP beads. PCR amplification was performed, and the ligation products were PCR amplified and product-purified using AMPure XP beads to generate the finished cDNA library (PE 150 bp). The cDNA library was sequenced on an Illumina HiSeq 2500 sequencing platform.

The raw reads were generated by the high-throughput Illumina sequencing platform, followed by a stringent filtering process to obtain clean data. These included the removal of raw reads containing adapter-ligated contaminants, reads with more than 10% of N (unknown nucleotides), and low-quality reads (Q-value < 20). The remaining high-quality clean reads were mapped back to the assembled full-length transcripts of SMRT-Seq using Bowtie2 v2.3.5 [32]. Finally, the number of clean reads in each sample was mapped to each transcript and calculated using RSEM v1.3.1 [33].

#### 2.3.2. Differential Expression Analysis

Relative expression levels of transcripts were standardized based on the RSEM-counted number of fragments per kilobase per million fragments mapped (FPKM) formula. Statistical differential gene expression analysis among experimental groups was performed using DESeq2 assay [34]. Subsequently, the differentially expressed genes (DEGs) were screened through the threshold values of false discovery rate (FDR) < 0.05 and |log2 (fold-change)| > 1.

#### 2.3.3. STEM Analysis

We used the STEM program [35] to identify significant temporal expression profiles and the genes associated with them based on time-series and gradient temperature changes. Normalized gene expression data of FPKM were used to cluster genes by expression profile. The gene expression profiles that significantly correlated with the temperature gradient changes were obtained under the following standard conditions: the data preprocessing was normalized by transforming the data by log2 algorithm [36], the *p*-value of the significant profile less than 0.05 was considered to be statistically significant, the minimum correlation was set to 0.7, and the maximum unit change in model profiles between time points was set equal to 2 [37]. The gene profiles that significantly correlated with the temperature gradient were used for subsequent GO and KEGG enrichment analyses conducted using the DIAMOND program, which was based on hyper-geometric distribution. The GO terms and KEGG pathways were statistically considered as significant enrichment functional clusters when the FDR ≤ 0.05.

#### 2.3.4. WGCNA Analysis

Based on gene expression matrix data, a gene co-expression module was constructed using the WGCNA functional package [38]. Genes with similar expression patterns were clustered in a module. Based on the correlation coefficient R and the corresponding *p*-value between the module eigengene (ME) values and temperatures, the relationships between these modules and cold stress (specific traits or phenotypes) were analyzed [37]. Subsequently, the modules with |R| > 0.70 and *p* < 0.001 were selected as temperature-related specific modules [39]. The function enrichment of the GO item and KEGG pathway for genes in temperature-related specific modules were analyzed using the DIAMOND program.

Hub genes were determined following the connectivity of the co-expression network. The data sets of protein–protein interaction (PPI) were sorted through a descending weight value. The first 200 protein interaction pairs were used to draw the PPI diagram using Cytoscape v3.7.2 software [40]. The PPI nodes with the highest connectivity were considered the ‘hub genes’.

### 2.4. Quantitative Real-Time PCR (qRT-PCR) Validation

We randomly screened eight DEGs and analyzed them using qRT-qPCR to examine the reliability of the RNA-seq technique. The 18S rRNA was used as the internal reference gene for data normalization. Gene-specific primers (Table 1) were designed based on the target gene sequences using Primer-Premier 5 software (Premier Biosoft Interpairs, Palo Alto, CA, USA). Total RNA was extracted from the CT, LT20, and LT12 groups, and 1000 ng RNA was used for reverse transcription. Reverse transcription was performed using the TureScript 1st Stand cDNA Synthesis Kit (Aidlab, Beijing, China) following the instructions from the manufacturer.

Further, qRT-PCR amplifications were conducted in 20 μL reaction volumes consisting of 7.8 μL RNase-free water, 10 μL 2× SYBR^®^ Green Supermix reagent (SYBR, Dalian, Liaoning, China), 1 μL cDNA, and 0.6 μL of each primer (10 mM). Real-time PCR was amplified in an ANALYTIKJENA QTOWER 2.2 (Analytik Jena AG, Jena, Germany). The qRT-PCR conditions were as follows: preheating at 95 °C for 5 min; followed by 40 cycles of 95 °C for 15 s; and 60 °C for 30 s. There were 9 samples in three groups, and each sample repeated three reactions. Each plate contained a NTC (no template control) for detecting potential contamination in the reaction mixture. The amplification curves were used to judge the range of circle threshold (CT) and the stable expression of 18S in all samples. Melt curves and standard curves were used to judge the specificity and amplification efficiency of primers, respectively. The relative expression of target genes was calculated using the comparative CT method (2^−^^ΔΔCT^) [41].

## 3. Results

### 3.1. PacBio SMRT-Seq Sequencing, Assembly, and Functional Annotation

In total, 37.15 Gbp polymerase reads were generated through SMRT-Seq sequencing. After discarding traces of PacBio adapters, 34.93 Gbp sub-reads with an average length of 1190 bp and their N50 of 1389 bp were generated. Total bases of CCS were 526,739,880 bp with an N50 of 1676 bp, of which 369,278,340 bp were 301,448 FLNC reads with a total mean length of 1226 bp and N50 of 1440 bp. The total average length of corrected transcripts bases was 16,191,669 bp. Finally, a total of 15,311,361 bp full-length transcripts were obtained, and their mean length and N50 values were 1388 and 1774 bp, respectively. The resulting full-length transcripts were used as reference sequences for further RNA-Seq analysis.

**Figure 1:**
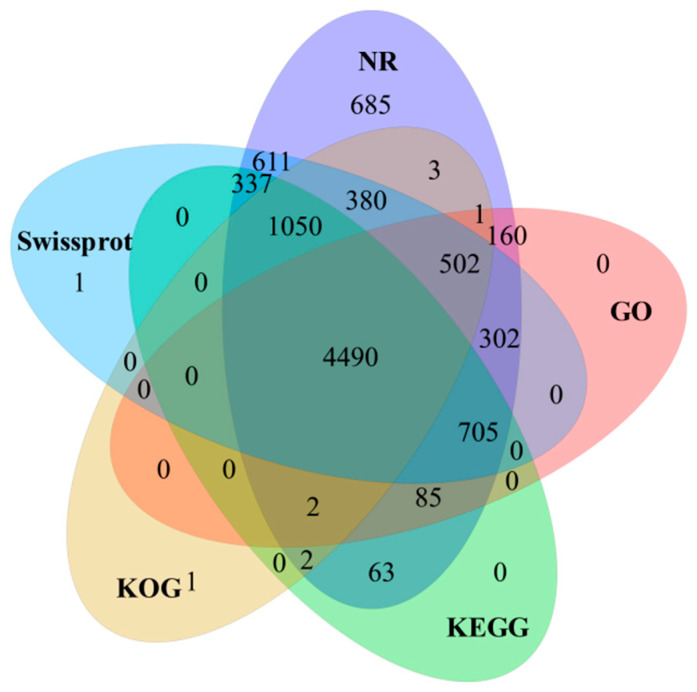
Comprehensive functional annotation of full-length transcripts against NR, Swissprot, KOG, GO, and KEGG databases.

Comprehensive functional annotation of 11,033 full-length transcripts was performed by NR, SWISS-PROT, KOG, GO, and KEGG databases (Figure 1). A total of 9380 (85.02%) full-length transcripts were successfully annotated (E-value < 1 × 10^−10^) using at least one of the five databases. Among them, 9378 (85.00%), 8378 (75.94%), 6431 (58.29%), 6247 (56.62%), and 6734 (61.04%) full-length transcripts were annotated based on alignment to NR, SwissProt, KOG, GO, and KEGG databases, respectively. The remaining 1653 (14.98%) unannotated transcripts could represent other novel specific genes. In comparison to previously annotated fish transcripts in the NR database, our top five transcript hits included *Lates calcarifer* (16.3%), *Larimichthys crocea* (10.4%), *Epinephelus coioides* (9.0%), *Seriola dumerili* (8.7%), and *Seriola lalandi* (4.8%).

### 3.2. Illumina RNA-Seq Analysis

**Figure 2:**
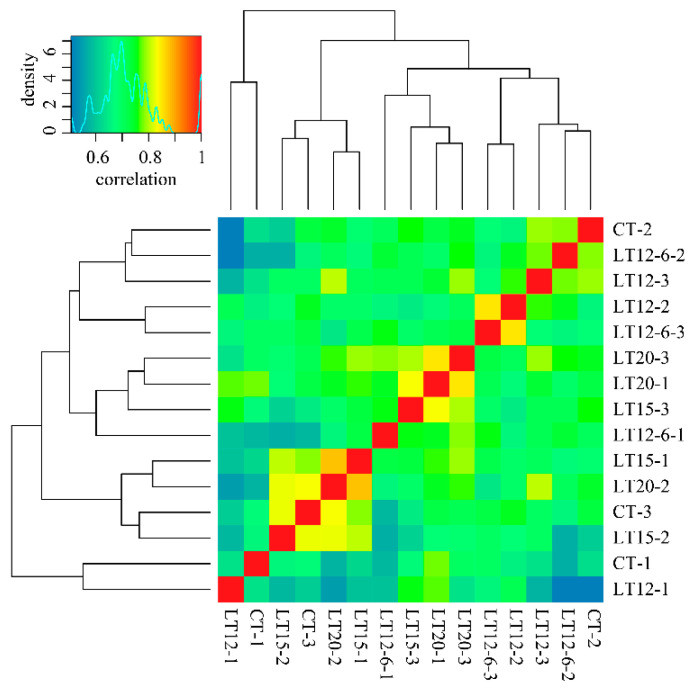
The correlation coefficient between samples.

The expression matrix data of all transcripts in samples were standardized by FPKM and used for correlation analysis and differential expression analysis. The correlation coefficients of three repeated experiments in each group mainly ranged from 0.6 to 0.9 (Figure 2). A total of 105.65 Gbps clean reads (Q30 > 92.49%) were obtained after filtering the 106.33 Gbps ambiguous raw reads. As illustrated in Table 2, its GC contents ranged from 48.87% to 50.06%, and the error rate per sample was below 0.05%. Among the 15 samples, the base number of clean reads ranged from 21,798,947 to 27,220,097, of which a range between 75.75~79.59% reads was successfully mapped to the full-length transcripts obtained using SMRT-Seq. Of these mapped reads, the multiple mapping rate ranged from approximately 64.21~68.48%. The number of differentially expressed transcripts in each sample was 8405~8647.

### 3.3. DEGs Identification

**Figure 3:**
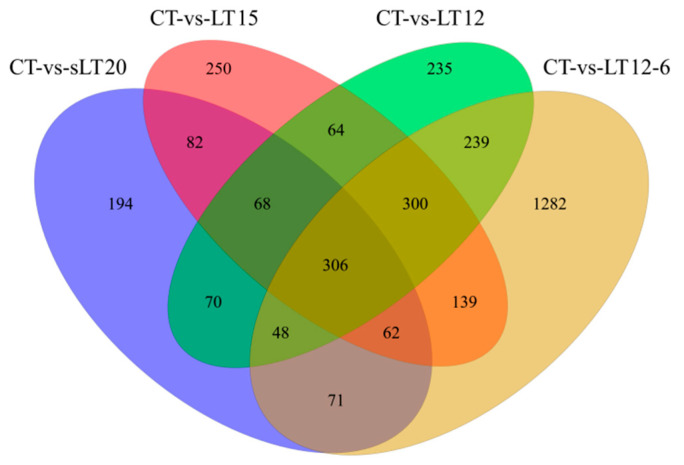
Venn diagrams of differentially expressed genes (DEGs) in different groups.

The DEGs were identified between different temperature experimental groups. As demonstrated in Figure 3, there were 901 (496 up- and 405 down-regulated), 1271 (725 up- and 546 down-regulated), 1330 (787 up- and 543 down-regulated), and 2447 (1243 up- and 1204 down-regulated) genes shown to be differentially expressed in comparison to CT vs. LT20, CT vs. LT15, CT vs. LT12, and CT vs. LT12-6, respectively (Tables S1–S4). According to the comparative analysis between control group and low temperature groups, 3410 differentially expressed genes were screened. The Venn diagram showed that four comparisons shared 306 DEGs. Notably, in the comparisons of LT20 vs. LT15, LT20 vs. LT12, and LT20 vs. LT12-6, there were a total of 431 (230 up- and 201 down-regulated), 799 (471 up- and 328 down-regulated), and 1956 (1064 up- and 892 down-regulated) DEGs, respectively. In addition, 668 (344 up- and 324 down-regulated), 1465 (762 up- and 703 down-regulated), and 1120 (577 up- and 543 down-regulated) DEGs were identified in comparison to LT15 vs. LT12, LT15 vs. LT12-6, and LT12 vs. LT12-6, respectively. These results showed that more DEGs were screened along with the decrease of water temperature and the extension of cold stress time.

### 3.4. STEM Analysis

**Figure 4:**
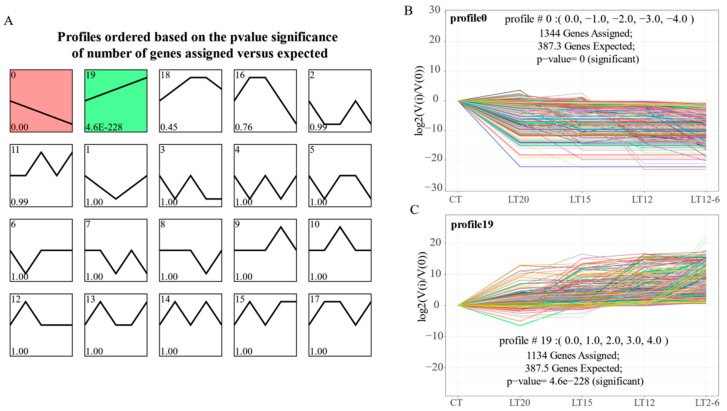
Short time-series expression profiles based on the temperature gradient. (**A**) Twenty time-series expression profiles; (**B**) The expression trend of genes in profile 0; (**C**) The expression trend of genes in profile 19. The horizontal axis represents temperature gradient, and the longitude axis represents the level of gene expression (up-or down-regulation).

Statistically significant *p*-values were found in profiles 0 and 19, and they had more genes than other profiles (Figure 4A). As shown in Figure 4B, profile 0 had 1344 consistently down-regulated genes, and most of them were down-regulated at the LT20 group. In contrast, a total of 1134 genes were consistently up-regulated in profile 19 (Figure 4C), and most of them were up-regulated at the LT20 group. Subsequently, some genes maintained original expression levels after the first down- or up-regulation, whereas others were continuously down- or up-regulated in all gradient nodes.

**Figure 5:**
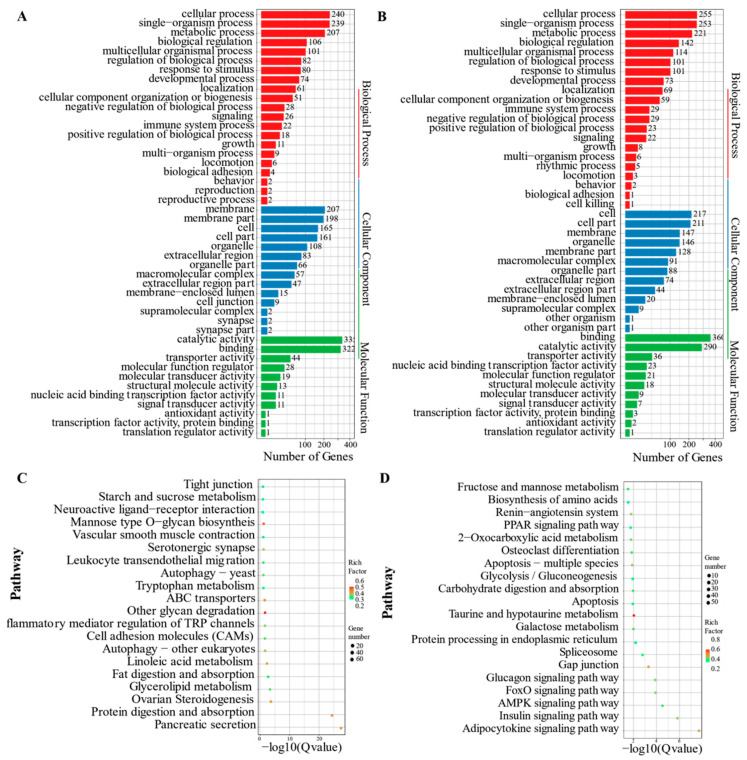
GO and KEGG enrichment analysis of profile 0 and profile 19. (**A**) GO enrichment of profile 0; (**B**) GO enrichment of profile 19; (**C**) KEGG enrichment of profile 0; (**D**) KEGG enrichment of profile 19. Gene_number: The number of DEGs in the current pathway. RichFactor: The ratio of the DEG number and the number of genes annotated in the pathway. Q value: The corrected *p*-value using the FDR method.

The KEGG pathway and GO enrichment analyses were performed on profiles 0 and 19 to assess the key biological processes and pathways linked with cold stress. As demonstrated in Figure 5, the main GO or KEGG terms observed in profiles 0 and 19 were similar. In the GO enrichment analysis (Figure 5A,B), different terms were represented. They included cellular process, single-organism process, metabolic process, biological body process, membrane, cell, cell part in cellular components, catalytic activity, and molecular function binding. On the other hand, the main enrichment pathways in KEGG were illustrated in Figure 5C,D. They included different signaling pathways, such as insulin, AMPK, FOXO, glucagons, and adipocytokine. The others included pancreatic secretion, protein digestion and absorption, ovarian steroidogenesis, and linoleic acid metabolism.

### 3.5. WGCNA

**Figure 6:**
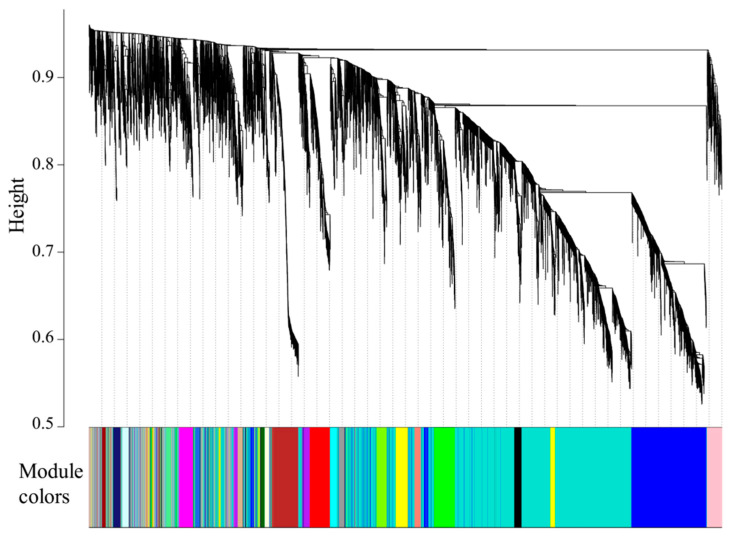
Cluster dendrogram of genes showing 23 modules identified by WGCNA. The cluster tree branches represent genes, and the genes with similar expressions were clustered in the same module (color).

**Figure 7:**
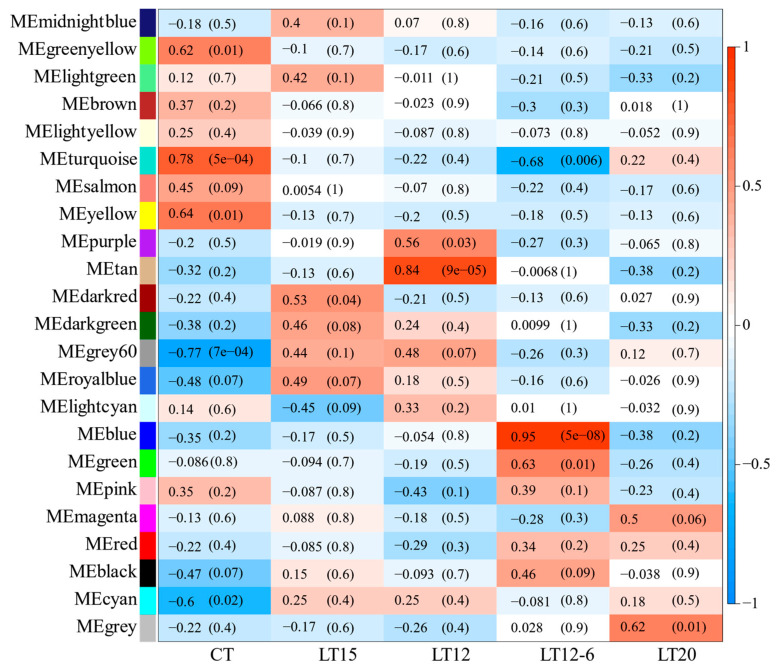
Module-traits relationships. The horizontal axis represents trait groups (temperature gradient), and the longitude axis shows 23 modules. The color scale on the right shows module-trait (temperature) correlation from −1 (blue) to 1 (red), indicating low to high correlations.

In this study, 23 modules were obtained based on the co-expression network analysis of 8826 mRNAs (Figure 6), and the gene number in each module ranged from 55 to 3412 (Table 3). Among the 23 modules, only three were significantly associated with different temperatures. As shown in Figure 7, ME turquoise, tan, and blue modules were positively correlated with CT (R = 0.78, *p* = 5 × 10^−4^), LT12 (R = 0.84, *p* = 9 × 10^−5^), and LT12-6 (R = 0.95, *p* = 5 × 10^−8^) experimental groups, respectively. Of note, the number of genes in the ME tan module (133) and blue module (1343) was considerably lower than those in the turquoise module (3412).

**Figure 8:**
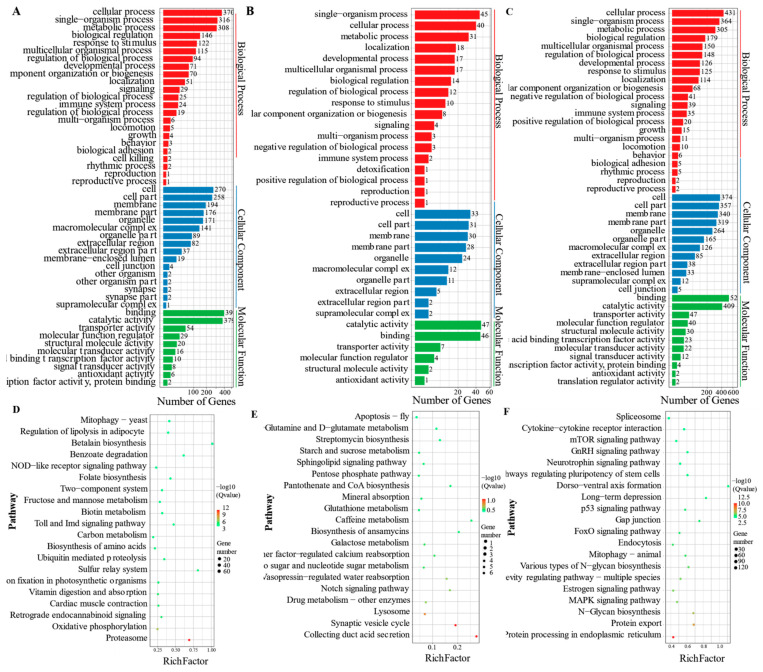
Enrichment analysis of temperature-related specific modules. (**A**–**C**) showed GO enrichment analysis of blue, tan, and turquoise modules, respectively. (**D**–**F**) showed KEGG enrichment analysis of blue, tan, and turquoise modules, respectively.

The GO and KEGG enrichment analyses were performed on the above three modules to further explore the functions of genes in the temperature-related specific modules. The main GO enrichment terms (Figure 8A–C), similar to the three modules, included cellular process, single-organism process, metabolic process, cell, cell part, membrane, binding, and catalytic activity. The KEGG enrichment analysis (Figure 8D,E) showed that both tan and blue modules are mainly enriched in the adipocytokine, glucagon, FOXO, insulin, and apelin signaling pathways. Compared with the turquoise module (Figure 8F), many biological processes and pathways were inhibited under low-temperature conditions. In brief, as the water temperature decreased, gene expression activity also decreased, and only key genes were expressed to resist cold stress.

**Figure 9:**
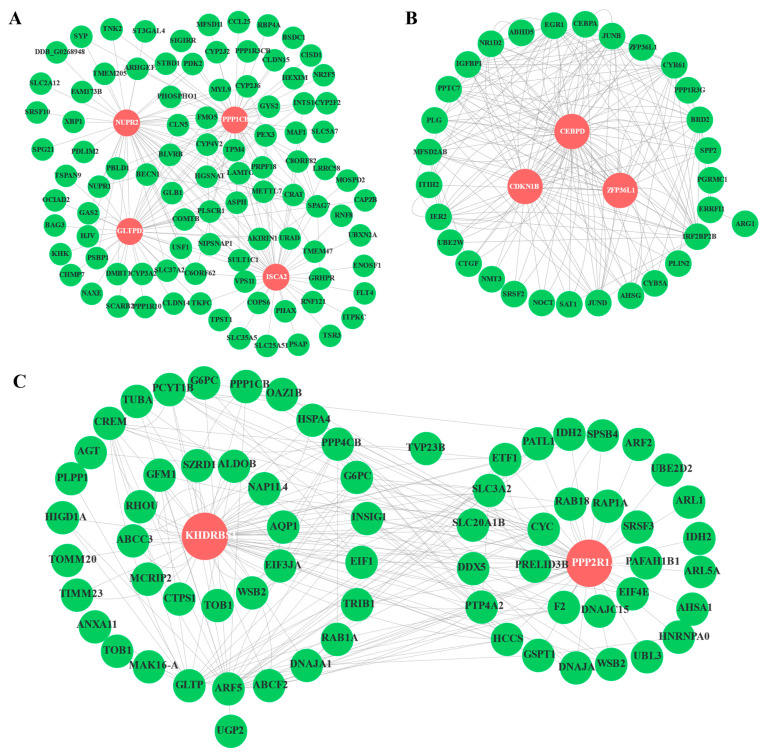
The PPI networks included turquoise, tan, and blue modules. (**A**) The PPI network of the turquoise module with 98 nodes and 200 edges; (**B**) The PPI network of ME tan module with 33 nodes and 200 edges; (**C**) The PPI network of ME blue module with 70 nodes and 200 edges. Hub genes were highlighted with red color as the background, and other genes were shown in green circles.

We further analyzed the PPI network analysis to find the hub and key genes with vital regulatory effects in the three modules. Here, the turquoise module possessed 98 nodes and four hub genes (ppp1cb, NUPR2, GLTPD2, ISCA2); the blue module contained 70 nodes and two hub genes (KHDRBS1, PPP2R1A); whereas the tan module only included 33 nodes and three hub genes (Cebpd, CDKN1B, Zfp36l1) (Figure 9). By calculating the connectivity and sum comparison of each edge, hub genes were identified that had the highest connectivity within these three modules.

### 3.6. Validation of DEGs Using qRT-PCR

**Figure 10:**
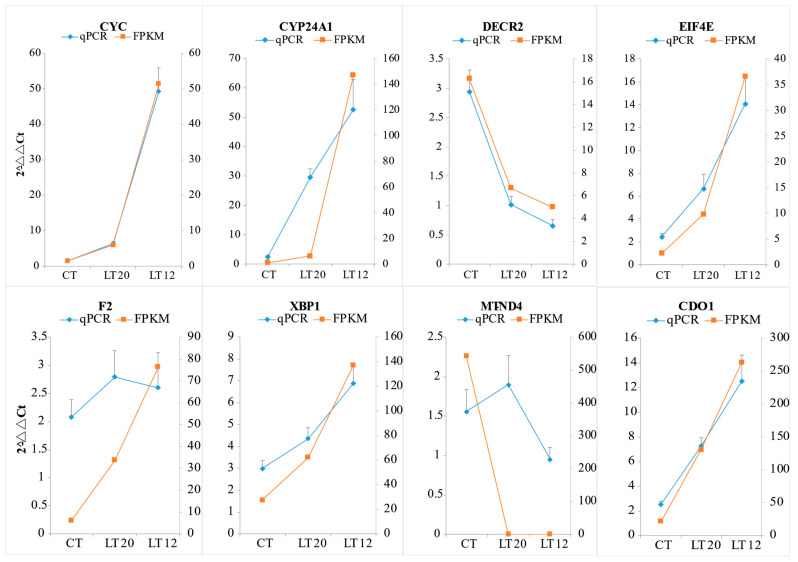
Validation of RNA-Seq results using RT-qPCR. The horizontal axis represents groups, and the longitude axis represents the relative expression level.

The relative expression profiles of eight DEGs were validated through qRT-PCR. As demonstrated in Figure 10, CYC, CYP24A1, EIF4E, F2, XBP1, and CDO1 were up-regulated, whereas DECR2 and MT-ND4 were down-regulated. Notably, all the expression profiles in the eight genes were consistent with the RNA-Seq results.

## 4. Discussion

Based on transcriptomic analyses, this study screened 3271 cold-related candidate genes and two low-temperature specific modules (ME tan and blue module) and identified two significant expression gene sets (profiles 0 and 19) in the pearl gentian grouper. Besides, we revealed some true key genes (e.g., ALDOB, PCK, FBP, G6PC, CPT1A, CDKN1B, and PPARα) and biological pathways (e.g., glycolysis/gluconeogenesis, AMPK signaling pathway, FOXO signaling pathway, insulin signaling pathway, and glucagon signaling pathway) that are closely related to cold stress (Table 4). These results provided reliable clues and new findings to explore the molecular regulation mechanisms of the pearl gentian grouper under cold stress.

### 4.1. Metabolic Processes Associated with Cold Stress

**Figure 11:**
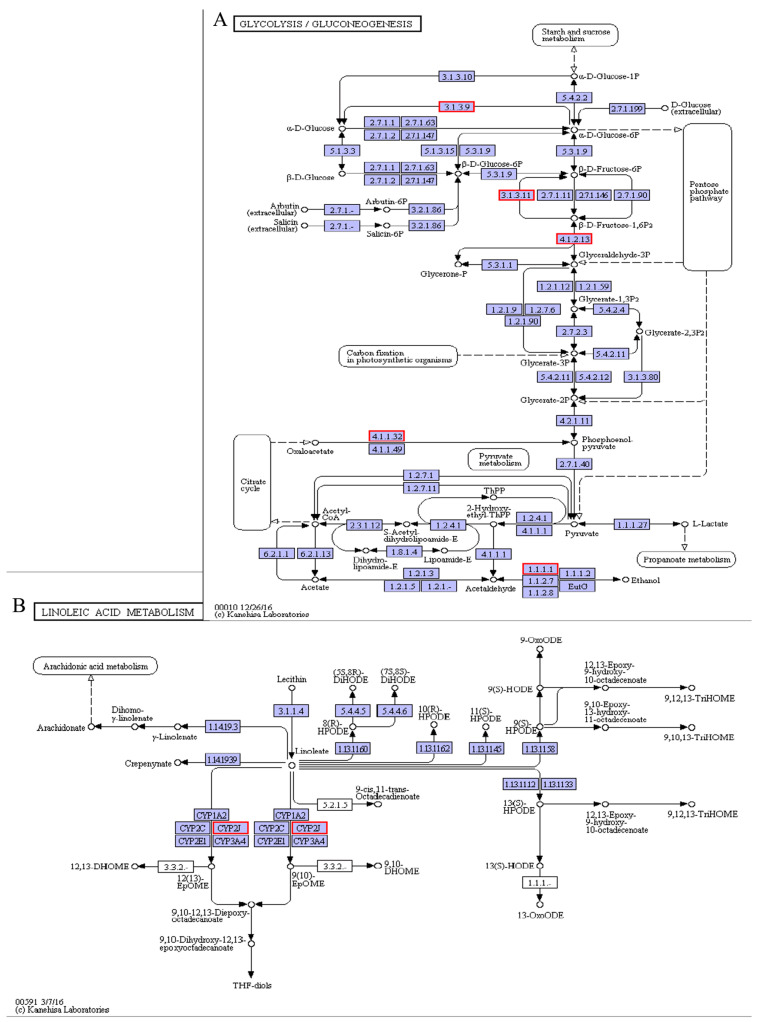
Glycolysis/gluconeogenesis pathway (**A**) and linoleic acid metabolism pathways (**B**).

Energy maintenance is a requirement that is particularly important for animals to resist cold stress. Many marine fishes use carbohydrate and lipid metabolism pathways to balance energy and withstand cold stress [42,43]. During carbohydrate metabolism, including glycolysis/gluconeogenesis (Figure 11A) and fructose/mannose metabolism, some important genes were significantly enriched in the pearl gentian grouper. Compared with the control group, the fructose-bisphosphate aldolase B (ALDOB, 4.1.2.13) gene was drastically down-regulated, whereas the phosphoenolpyruvate carboxykinase (PCK1, 4.1.1.32), fructose-1,6-bisphosphatase 1 (FBP1, 3.1.3.11), fructose-1,6-bisphosphatase isozyme 2 (FBP2, 3.1.3.11), and glucose-6-phosphatase (G6pC, 3.1.3.9) genes were significantly up-regulated. Hepatic gluconeogenesis is an important process that maintains blood glucose levels during fasting, and it needs four key rate-limiting enzymes to function. Among the four enzymes, three of them, including PCK, FBP, and G6pC, have been explored [44]. The PCK1 gene catalyzes the irreversible cataplerotic conversion of oxaloacetate (OAA) to phosphoenolpyruvate (PEP) [45]. Thus, by up-regulating this gene in the pearl gentian grouper, we would increase PEP release and greatly promote the gluconeogenesis process, which also promotes glucose synthesis from lactate and other precursors under low-glucose conditions. On the other hand, the ALDOB efficiently synthesizes glyceraldehyde-3-phosphate and dihydroxyacetone phosphate from fructose-1,6-bisphosphate, and its down-regulation is associated with the accumulation of fructose-1,6-bisphosphate. Subsequently, the resultant fructose-1,6-bisphosphate could be hydrolyzed to fructose-6-phosphate by up-regulating FBP [46]. Then, fructose-6-phosphate could be converted to glucose-6-phosphate in the endoplasmic reticulum lumen, and the resultant glucose-6-phosphate is further hydrolyzed to glucose by up-regulating the G6pC gene [47]. Taken together, by down-regulating the ALDOB gene and up-regulating the rate-limiting enzyme (PCK1, FBP1, FBP2, and G6pC) genes, it is therefore conducive to promote glucose synthesis. Hence, providing energy for cell survival ultimately increases the ability of the pearl gentian grouper to survive in low-temperature.

Membrane lipid fluidity is vital for many physiological functions of animals. Its degree is mainly determined by calculating the ratio of unsaturated to saturated fatty acids and cholesterol ratio to phospholipids [48,49]. Cold stress could alter the animal cell membrane fluidity and induce tissue damage [50]. In the pearl gentian grouper, we detected that Cytochrome P450 2J1 (CYP2J1), Cytochrome P450 2J2 (CYP2J2), and Cytochrome P450 2J6 (CYP2J6) genes in linoleic acid metabolism pathways were significantly down-regulated under cold stress (Figure 11B). As a subfamily of the cytochrome P450 (CYP) enzyme, the CYP2J catalyzes the epoxidation of arachidonic acid to epoxyeicosatrienoic acids (EETs) and linoleic acid to epoxyoctadecenoic acids (EpOMEs) [51]. By down-regulating the CYP2J enzyme, there would be a decrease in the epoxidation of arachidonic and linoleic acids, thereby increasing the unsaturated fatty acids content. Generally, higher unsaturated fatty acids content indicates a greater fluidity of membrane lipids [52]. Hence, the down-regulation of CYP2J genes helped maintain cell membrane fluidity and normal physiological functions of the pearl gentian grouper under cold stress. Similar CYP gene expression trends were observed in cold-stress exposed *Epinephelus coioides* [53].

### 4.2. Response of Signal Transductions to Cold Stress

Signal transduction is critical for the survival and adaption of fish under cold stress acclimation [54]. On the other hand, the CAMP and MAPK signaling pathways were considered essential signals in fishes under low-temperature environments [21]. Our analyses observed that in the pearl gentian grouper, both the AMPK and FOXO signaling pathways were mainly enriched under cold stress conditions.

**Figure 12:**
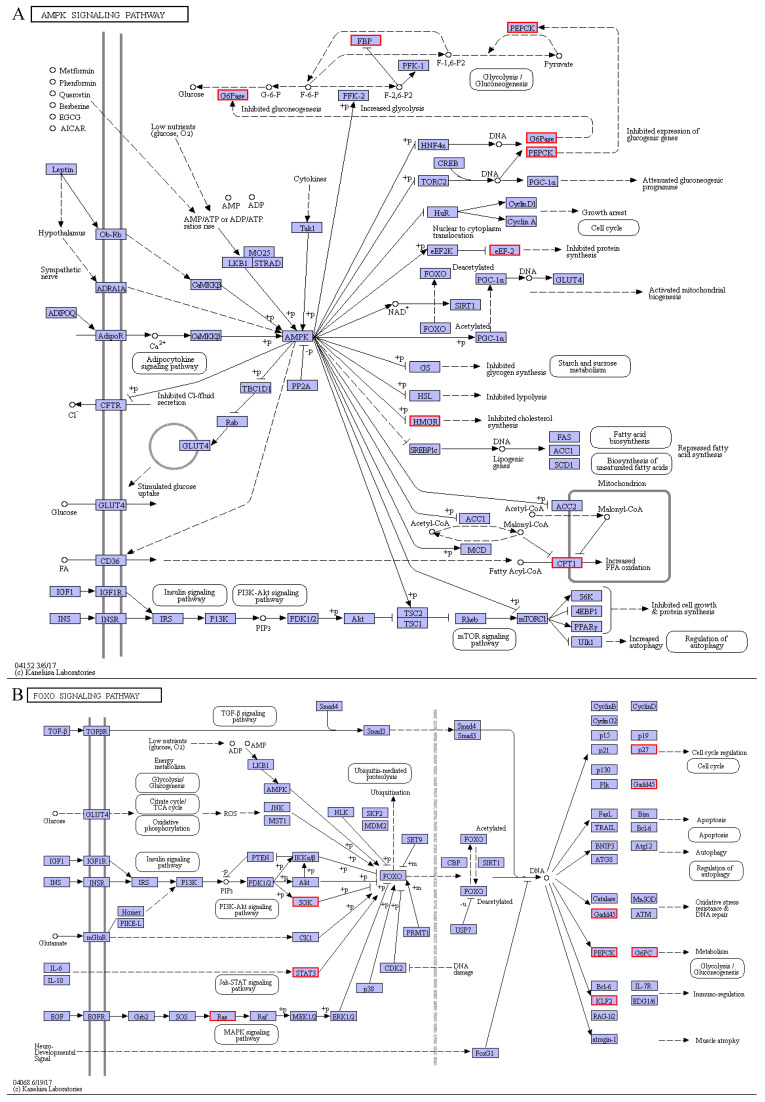
AMPK signaling pathway (**A**) and FOXO signaling pathway (**B**).

The AMPK pathway is a crucial cellular regulator of glucose and lipid metabolisms and a key regulator of cellular energy homeostasis [55]. In this study, the AMPK signaling pathway further demonstrates that cold stress promotes gluconeogenesis by up-regulating the expression levels of three key rate-limiting enzymes (G6PC, PCK, and FBP) in the pearl gentian grouper (Figure 12A). Moreover, cold stress promotes the oxidation of long-chain fatty acids, another major pathway for energy production in animals, through the up-regulation of carnitine o-palmitoyltransferase 1a (CPT1A, liver isoform) in the AMPK signaling pathway [5]. Since CPT1A is the rate-limiting enzyme in mitochondrial fatty acid β-oxidation, it catalyzes long-chain fatty acid transfer to the mitochondrion [56]. Therefore, detecting the up-regulation of the CPT1A in our AMPK signaling pathway indicates an increase in mitochondrial fatty acyl-CoA content and further upregulated fatty acids β-oxidation. Thus, increasing ATP production protects the pearl gentian grouper against cold stress. Taken together, these results suggest that the AMPK signaling pathway increases energy metabolism to maintain energy homeostasis in the pearl gentian grouper under cold stress conditions.

In the AMPK signaling pathway, we also observed that cholesterol synthesis is regulated by the up-regulation of the 3-hydroxy-3-methylglutaryl-coenzyme A reductase (HMGCR) gene. In the liver, it is the rate-limiting enzyme during cholesterol synthesis that catalyzes the conversion of (3S)-hydroxy-3-methylglutaryl coenzyme A (HMG-CoA) to mevalonate (MVA) [57]. The up-regulation of the HMGCR gene detected here could promote cholesterol synthesis, which is favorable to maintain the cholesterol homeostasis of the pearl gentian grouper under cold stress. Moreover, our RNA-seq analysis showed that some other genes like 7-dehydrocholesterol reductase (DHCR7), 1,25-dihydroxyvitamin D (3) 24-hydroxylase (CYP24A1), sterol O-acyltransferase 1 (SOAT1), and delta (24)-sterol reductase (DHCR24) in the steroid biosynthesis pathway were also up-regulated. This result provides additional evidence on the cold-dependent regulation during cholesterol synthesis. Cholesterol is important and indispensable in the functioning of animal cell membranes. Its content can effectively regulate permeability, molecular order, elasticity, orientation, and intermolecular spacing of lipid membranes, closely related to cell membrane adaptation [23]. Based on the above analyses, we conclude that the changes in cell lipid membranes caused by increased cholesterol content could be an important molecular mechanism in response to cold stress in the pearl gentian grouper.

Besides, we found that cold stress significantly up-regulated the expression levels of three key genes in three important FOXO signaling pathways, including serine/threonine-protein kinase SGK1 of the PI3K-Akt signaling pathway, signal transducer and activator of transcription 1α/β (STAT1) of the Jak-STAT signaling pathway, and GTPase KRAS of the MAPK signaling pathway. It has been confirmed that the FOXO signaling pathway implicates extensive cellular functions of animals, and many signal transduction pathways usually converge in FOXO [58]. The above three genes and their pathways play crucial roles in intracellular signal transduction pathways, which participate in various cellular processes, like stress, differentiation, proliferation, apoptosis, and immunity [59,60,61]. Activating the FOXO and the AMPK signaling pathways further increases gluconeogenesis-related genes (PCK1, PCK2, and G6PC) in the pearl gentian grouper. This suggests that the FOXO signaling pathway involves regulating glucose homeostasis and energy metabolism in the pearl gentian grouper under cold stress conditions.

Moreover, activating the FOXO signaling pathway up-regulates the expression levels of several genes related to cell cycle (cyclin-dependent kinase inhibitor p27, CDKN1B), oxidative stress resistance and DNA repair (growth arrest and DNA-damage-inducible protein 45β, GADD45β), and immuno-regulation (krueppel-like factor 2, KLF2). Based on the WGCNA analysis, this study established that the CDKN1B gene is a hub gene in the ME tan module, a low-temperature specific module in the pearl gentian grouper. Subsequently, this gene was demonstrated to inhibit DNA synthesis during G1 and S phases and negatively regulates the cell cycle by preventing the activation of cyclin-dependent-kinase 2 (CDK2) [62]. Therefore, we can infer that the up-regulation of the CDKN1B gene would slow cell cycle progression and hinder cell differentiation, which is useful in saving enough energy to resist low temperature for the pearl gentian grouper. The Gadd45 family (Gadd45a, Gadd45b, and Gadd45g), a stress sensor, is implicated in cell cycle arrest, DNA repair, cell survival, or apoptosis caused by environmental stress [63,64]. The up-regulation of the Gadd45b gene detected here suggests that continuous cold stress could induce cell cycle disorder, DNA damage, or cell apoptosis for the pearl gentian grouper. In summary, cold stress changed the expression levels of some genes related to energy metabolism, cell cycle, etc., which indicated that the FOXO signaling pathway could play an important role in resisting cold stress injuries in the pearl gentian grouper.

### 4.3. Endocrine System and Cold Stress

**Figure 13:**
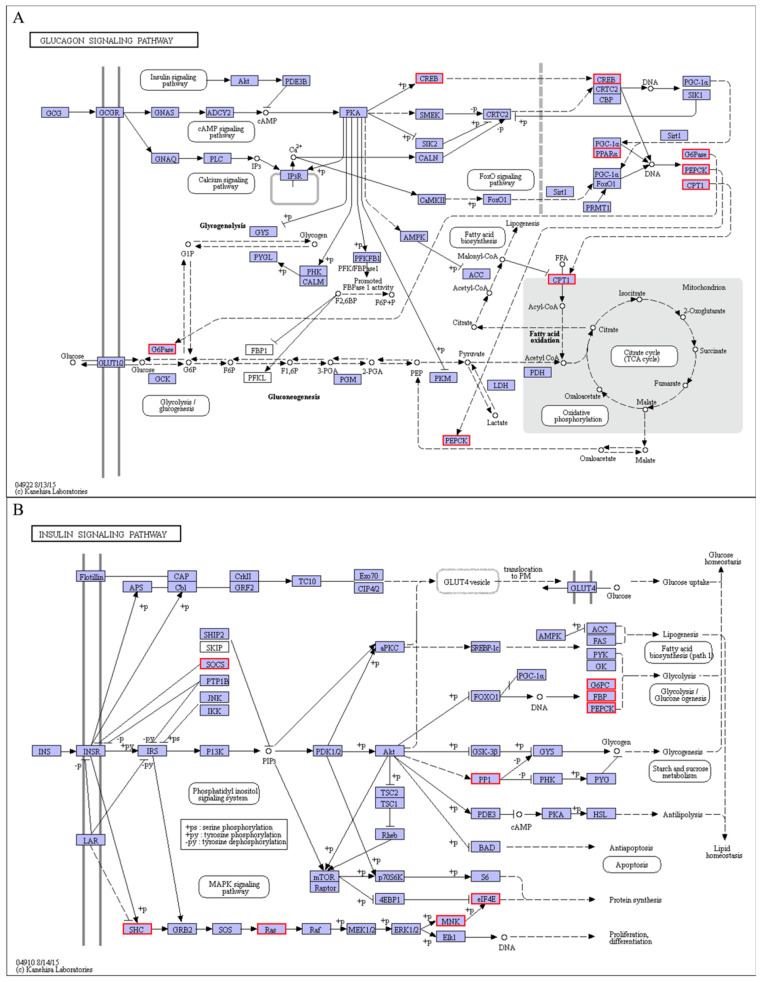
Glucagon signaling pathway (**A**) and insulin signaling pathway (**B**).

It is generally known that the endocrine hormones could control glucose concentration and regulate energy balance through an antagonistic effect of insulin against glucagon [44,65]. Therefore, the hormonal stress response levels in the endocrine systems could be applied to understand the coping mechanism of animals under cold stress [66]. We discovered that cold stress-activated the glucagon signaling pathways. Several genes, such as peroxisome proliferator-activated receptor alpha (PPARα) (Figure 13A) related to energy metabolism, were also significantly up-regulated. The PPARα gene is highly expressed in the liver tissues and is well known as a master regulator of lipid and glucose metabolism, specifically the fatty acid β-oxidation [67]. Besides, its activation in the pearl gentian grouper significantly up-regulates the expression of fatty acid and glucose metabolism genes, including CPT1, PCK1, PCK2, and G6PC. When combined with signal transduction responses and metabolic processes, we could suggest that cold stress stimulated the AMPK-PPARα-CPT1 signaling pathway and promotes the gluconeogenesis pathway, thereby maintaining glucose, lipid, and energy homeostasis in the pearl gentian grouper under cold stress conditions.

Additionally, in the glucagon signaling pathway, the cold stress condition was observed to activate the insulin signaling pathway and robustly induce the expression of suppressor of cytokine signaling 3 (SOCS3) and serine/threonine-protein phosphatase PP1-gamma catalytic subunit A (PPP1CC-A) (Figure 13B). The SOCS3 signaling pathway has been shown to inhibit the insulin signaling pathway [68]. Therefore, its up-regulation could inhibit insulin expression. Insulin concentration is inversely proportional to blood glucose levels [44]. In other words, the increased expression of SOCS3 in the pearl gentian grouper could promote an increase in its glucose production. On the other hand, the up-regulated expression of PPP1CC was reported to enhance glycogen synthase (GYS) activity and inhibit phosphorylase kinase (PHK) and glycogen phosphorylase (PYG) activities through dephosphorylation [44]. These activities increase glycogen synthesis rates and account for glucose transformation. In conclusion, SOCS3 and PPP1CC genes involved regulating blood glucose concentration of the pearl gentian grouper under cold stress conditions.

In this study, low temperature affects energy metabolism, lipid membranes, and protein metabolism of the pearl gentian grouper. Our results showed that several genes such as SHC-transforming protein 1 (SHC2), GTPase KRAS, MAP kinase-interacting serine/threonine-protein kinase 2 (MKNK2), eukaryotic translation initiation factor 4E (eIF4E) (Figure 13B), and eukaryotic elongation factor 2 (eEF2) (Figure 12A), related to protein metabolism were significantly up-regulated under cold stress conditions, promoting protein synthesis in the pearl gentian grouper. The increased capacity in protein synthesis could be relevant to metabolic adjustment in response to cold stress. It has been demonstrated that cold stress could cause oxidative stress and lead to protein damage in animal cells [69]. In contrast, the continuously synthesized proteins could replace damaged proteins and participate in cell metabolic regulation, which is vital for fish to adapt to cold stress [70]. Therefore, it can be inferred that continuous cold stress induces protein metabolism changes, which could involve the impairment of the cold-induced effect of the pearl gentian grouper.

Furthermore, several biological pathways, including platelet activation, complement and coagulation cascades, vascular smooth muscle contraction, cardiac muscle contraction, and serotonergic synapse pathways, were also detected based on our RNA-seq results that analyzed the cold stress responses of the pearl gentian grouper. The up-regulation or down-regulation of genes in these pathways could be physiologically adjusted to reduce damage and maintain the basic life functions of the pearl gentian grouper under cold stress. This suggests that the pearl gentian grouper should undergo some cellular processes under cold stress and resist cold stress by activating various pathways. However, the above pathways and their related genes were not successfully identified in our WGCNA analysis. Thus, future research needs to confirm whether these pathways and their related genes play a substantial role in the cold stress response of the pearl gentian grouper.

## 5. Conclusions

Based on liver transcriptome analyses, this study demonstrates that the cold-related genes of the pearl gentian grouper in response to cold stress are most significantly enriched through carbohydrate metabolism, lipid metabolism, signal transduction, and endocrine system pathways. These pathways divide into several other major categories, including energy metabolism, stress-induced cell membrane changes, and stress signals transduction. Moreover, we further screened out some core candidate genes closely related to cold stress in the above pathways, including PCK, ALDOB, FBP, G6pC, CPT1A, PPARα, SOCS3, PPP1CC, CYP2J, HMGCR, CDKN1B, and GADD45B. Among them, energy-related metabolic pathways and genes had higher expression levels under cold stress, suggesting that energy homeostasis plays a crucial role in the physiological adjustments of the pearl gentian grouper when exposed to the cold stress environment. The pathways and genes identified in this study extend our understanding of the mechanisms involved in the cold stress response in marine fishes and facilitate selective aquaculture breeding of cold-tolerant pearl gentian grouper.

## Figures and Tables

**Table 1:** The sequences of specific primers for RT-qPCR.

Primer Name	Forward Primer (5′ to 3′)	Reverse Primer (5′ to 3′)
18S rRNA	GACTCTGGCGTGCTAACTA	CAATCTCGTGTGGCTGAAC
Cysteine dioxygenase type 1 (CDO1)	CCACGGCAGTAGCATCCA	CCATTCAAACAGCGTCTCCTTC
Cytochrome c (CYC)	AGACACCCTGATGATTTACTTGGA	CGTTCGCCCTTCTTCTTGATG
1,25-dihydroxyvitamin D (3) 24-hydroxylase, mitochondrial (CYP24A1)	TACGCAGCCATCACAGAG	AATCTCCTTCAGCAACTTCCT
Peroxisomal 2,4-dienoyl-CoA reductase (DECR2)	CTGCTGTGGATGAGACACTGAGAG	GGCGTTGAAGGAAAGTGCTGATG
Eukaryotic translation initiation factor 4E (EIF4E)	GCTTTGGCTGCGATTATTGTTT	GGTCATTGTGTCTCTGTTGCTTA
Prothrombin (F2)	GCTGCTGTTCTCCTGCTGTT	CGACGGCTCCTGACCAAC
NADH-ubiquinone oxidoreductase chain 4 (MT-ND4)	ATAACCCAGCGAGGACCC	GTAATGGTAGGAGGTGGAGTGT
X-box-binding protein 1 (XBP1)	GGTGGTGGAGGAGGAGAC	GGAGGAGGTTTCGGAGAAGA

**Table 2:** Detail data stats of RNA-Seq.

Sample	Raw Reads	Raw Bases	Clean Reads	Clean Bases	Error (%)	Q20 (%)	Q30 (%)	GC (%)
CT-1	40,457,922	6.07 G	40,193,928	6.03 G	0.03	97.62	93.14	50.06
CT-2	44,821,082	6.72 G	44,525,618	6.68 G	0.03	97.54	93.01	49.11
CT-3	44,301,766	6.65 G	43,997,216	6.6 G	0.03	97.31	92.49	49.09
LT20-1	40,314,958	6.05 G	40,050,718	6.01 G	0.03	97.84	93.61	49.68
LT20-2	44,029,006	6.6 G	43,747,514	6.56 G	0.03	97.63	93.16	49.28
LT20-3	40,525,754	6.08 G	40,259,138	6.04 G	0.03	97.45	92.77	49.53
LT15-1	43,878,738	6.58 G	43,597,452	6.54 G	0.03	97.59	93.07	49.53
LT15-2	43,106,336	6.47 G	42,820,278	6.42 G	0.03	97.46	92.81	49.45
LT15-3	58,979,158	8.85 G	58,580,046	8.79 G	0.03	97.46	92.82	49.44
LT12-1	51,108,766	7.67 G	50,768,078	7.62 G	0.03	97.52	92.95	49.29
LT12-2	56,321,806	8.45 G	55,947,676	8.39 G	0.03	97.55	93.03	49.32
LT12-3	49,213,672	7.38 G	48,877,820	7.33 G	0.03	97.42	92.73	49.12
LT12-6-1	41,337,836	6.2 G	41,069,634	6.16 G	0.03	97.56	93.03	49.16
LT12-6-2	50,932,700	7.64 G	50,598,692	7.59 G	0.03	97.53	92.95	49.27
LT12-6-3	48,065,372	7.21 G	47,731,214	7.16 G	0.03	97.4	92.74	49.21

**Table 3:** Modules (23) of WGCNA represented by specific colors.

**Module Colors**	**Genes**	**Module Colors**	**Genes**	**Module Colors**	**Genes**
turquoise	3412	pink	204	lightcyan	86
blue	1343	magenta	198	grey60	77
grey	747	purple	175	lightgreen	73
brown	382	greenyellow	165	lightyellow	70
yellow	375	tan	133	royalblue	64
green	332	cyan	104	darkred	57
red	324	salmon	104	darkgreen	55
black	244	midnightblue	102		

**Table 4:** Key pathways and genes.

Transcripts	Genes	LogFC	KEGG Pathways
shibanyu_transcript_846	PCK1 (Phosphoenolpyruvate carboxykinase, cytosolic)	12.37	Glycolysis/Gluconeogenesis, AMPK signaling pathway, FoxO signaling pathway, Insulin signaling pathway, Glucagon signaling pathway
shibanyu_transcript_6873	ALDOB (Fructose-bisphosphate aldolase B)	−14.17	Glycolysis/Gluconeogenesis
shibanyu_transcript_9393	FBP1 (Fructose-1,6-bisphosphatase 1)	2.01	Glycolysis/Gluconeogenesis, AMPK signaling pathway, Insulin signaling pathway
shibanyu_transcript_9234	FBP2 (Fructose-1,6-bisphosphatase isozyme 2)	2.68	Glycolysis/Gluconeogenesis AMPK signaling pathway, Insulin signaling pathway
shibanyu_transcript_599	G6PC (Glucose-6-phosphatase)	1.02	Glycolysis/Gluconeogenesis, AMPK signaling pathway, FoxO signaling pathway, Insulin signaling pathway, Glucagon signaling pathway
shibanyu_transcript_1763	CYP2J1 (Cytochrome P450 2J1)	−1.13	Linoleic acid metabolism
shibanyu_transcript_511	CYP2J2 (Cytochrome P450 2J2)	−1.69	Linoleic acid metabolism
shibanyu_transcript_1230	CYP2J6 (Cytochrome P450 2J6)	−1.55	Linoleic acid metabolism
shibanyu_transcript_7206	CPT1A (Carnitine O-palmitoyltransferase 1, liver isoform)	4.73	AMPK signaling pathway, Glucagon signaling pathway
shibanyu_transcript_168	PCK2 (Phosphoenolpyruvate carboxykinase [GTP], mitochondrial)	1.39	AMPK signaling pathway, FoxO signaling pathway, Insulin signaling pathway, Glucagon signaling pathway
shibanyu_transcript_1860	HMGCR (3-hydroxy-3-methylglutaryl-coenzyme A reductase)	3.19	AMPK signaling pathway
shibanyu_transcript_6395	DHCR7 (7-dehydrocholesterol reductase)	1.41	Steroid biosynthesis
shibanyu_transcript_4825	CYP24A1 (1,25-dihydroxyvitamin D(3) 24-hydroxylase, mitochondrial)	7.53	Steroid biosynthesis
shibanyu_transcript_371	SOAT1 (Sterol O-acyltransferase 1)	2.42	Steroid biosynthesis
shibanyu_transcript_1553	DHCR24 (Delta(24)-sterol reductase)	1.63	Steroid biosynthesis
shibanyu_transcript_4386	SGK1 (Serine/threonine-protein kinase Sgk1)	2.95	FoxO signaling pathway
shibanyu_transcript_397	STAT1 (Signal transducer and activator of transcription 1-alpha/beta)	1.50	FoxO signaling pathway
shibanyu_transcript_4950	CDKN1B (Cyclin-dependent kinase inhibitor 1B)	2.53	FoxO signaling pathway
shibanyu_transcript_5980	GADD45B (Growth arrest and DNA damage-inducible protein GADD45 beta)	3.23	FoxO signaling pathway
shibanyu_transcript_639	PPARα (Peroxisome proliferator-activated receptor alpha)	3.62	Glucagon signaling pathway
shibanyu_transcript_1906	SOCS3 (Suppressor of cytokine signaling 3)	3.16	Insulin signaling pathway
shibanyu_transcript_2800	PPP1CC-a (Serine/threonine-protein phosphatase PP1-gamma catalytic subunit A)	4.01	Insulin signaling pathway
shibanyu_transcript_229	SHC1 (SHC-transforming protein 1)	1.04	Insulin signaling pathway
shibanyu_transcript_8219	KRAS (GTPase KRas)	1.45	Insulin signaling pathway, FoxO signaling pathway
shibanyu_transcript_37	MKNK2 (MAP kinase-interacting serine/threonine-protein kinase 2)	2.55	Insulin signaling pathway
shibanyu_transcript_3250	EIF4E (Eukaryotic translation initiation factor 4E)	4.14	Insulin signaling pathway
shibanyu_transcript_4069	EEF2 (Elongation factor 2)	13.22	AMPK signaling pathway

Note: Name of transcript: grouper in Chinese (“shibanyu”) + “_” + “transcript” + “_” + Serial number (“xxxx”).

## Data Availability

PacBio and Illumina sequencing raw reads data and transcripts sequences have been uploaded to NCBI SRA database, item number is: PRJNA719178.

## Supplementary Materials

The following are available online at https://www.mdpi.com/article/10.3390/ani11061745/s1, Table S1, Table S2, Table S3, Table S4: Differentially expressed genes detail information in comparison to CT vs. LT20, CT vs. LT15, CT vs. LT12, and CT vs. LT12-6, respectively.

## References

[1] Donaldson M.R., Cooke S.J., Patterson D.A., Macdonald J.S. (2008). Cold shock and fish. J. Fish Biol..

[2] Samaras A., Papandroulakis N., Costari M., Pavlidis M. (2016). Stress and metabolic indicators in a relatively high (European sea bass, *Dicentrarchus labrax*) and a low (meagre, *Argyrosomus regius*) cortisol responsive species, in different water temperatures. Aquac. Res..

[3] Long Y., Li L., Li Q., He X., Cui Z. (2012). Transcriptomic characterization of temperature stress responses in larval zebrafish. PLoS ONE.

[4] Fan X., Qin X., Zhang C., Zhu Q., Chen J., Chen P. (2019). Metabolic and anti-oxidative stress responses to low temperatures during the waterless preservation of the hybrid grouper (*Epinephelus fuscogutatus* ♀ × *Epinephelus lanceolatus* ♂). Aquaculture.

[5] Lu D.L., Ma Q., Sun S.X., Zhang H., Chen L.Q., Zhang M.L., Du Z.Y. (2019). Reduced oxidative stress increases acute cold stress tolerance in zebrafish. Comp. Biochem. Physiol. Part A Mol. Integr. Physiol..

[6] Wang Q., Tan X., Jiao S., You F., Zhang P.J. (2014). Analyzing cold tolerance mechanism in transgenic zebrafish (*Danio rerio*). PLoS ONE.

[7] Wentworth S.A., Thede K., Aravindabose V., Monroe I., Thompson A.W., Molyneaux N., Owen C.L., Burns J.R., Vicente A.G., Garvin J.L. (2018). Transcriptomic analysis of changes in gene expression of immune proteins of gill tissue in response to low environmental temperature in fathead minnows (*Pimephales promelas*). Comp. Biochem. Physiol. Part D Genom. Proteom..

[8] Nitzan T., Kokou F., Doron-Faigenboim A., Slosman T., Biran J., Mizrahi I., Zak T., Benet A., Cnaani A. (2019). Transcriptome analysis reveals common and differential response to low temperature exposure between tolerant and sensitive blue tilapia (*Oreochromis aureus*). Front. Genet..

[9] Chu T., Liu F., Qin G., Zhan W., Wang M., Lou B. (2020). Transcriptome analysis of the *Larimichthys polyactis* under heat and cold stress. Cryobiology.

[10] Wen X., Hu Y., Zhang X., Wei X., Wang T., Yin S. (2019). Integrated application of multi-omics provides insights into cold stress responses in pufferfish *Takifugu fasciatus*. BMC Genom..

[11] Mininni A.N., Milan M., Ferraresso S., Petochi T., Di Marco P., Marino G., Livi S., Romualdi C., Bargelloni L., Patarnello T. (2014). Liver transcriptome analysis in gilthead sea bream upon exposure to low temperature. BMC Genom..

[12] Lin J., Shi X., Fang S., Zhang Y., You C., Ma H., Lin F. (2019). Comparative transcriptome analysis combining SMRT and NGS sequencing provides novel insights into sex differentiation and development in mud crab (*Scylla paramamosain*). Aquaculture.

[13] Huang B., Rong H., Ye Y., Ni Z., Xu M., Zhang W., Xu L. (2020). Transcriptomic analysis of flower color variation in the ornamental crabapple (*Malus spp.*) half-sib family through Illumina and PacBio Sequel sequencing. Plant Physiol. Biochem..

[14] Sharon D., Tilgner H., Grubert F., Snyder M. (2013). A single-molecule long-read survey of the human transcriptome. Nat. Biotechnol..

[15] Li Y., Fang C., Fu Y., Hu A., Li C., Zou C., Li X., Zhao S., Zhang C. (2018). A survey of transcriptome complexity in *Sus scrofa* using single-molecule long-read sequencing. DNA Res..

[16] Zhang Z., Yang Z., Ding N., Xiong W., Zheng G., Lin Q., Zhang G. (2018). Effects of temperature on the survival, feeding, and growth of pearl gentian grouper (female *Epinephelus fuscoguttatus* × male *Epinephelus lanceolatus*). Fish. Sci..

[17] Fan B., Liu X.C., Meng Z.N., Tan B.H., Wang L., Zhang H.F., Zhang Y., Wang Y.X., Lin H.R. (2014). Cryopreservation of giant grouper *Epinephelus lanceolatus* (Bloch, 1790) sperm. J. Appl. Ichthyol..

[18] Zhou Y., Han Y.L., Luo J., Wang X., Chen G.H. (2017). MSAP analysis of DNA methylation of *Epinephelus malabaricus*, *Epinephelus malabaricus* and their hybrid offspring. Nat. Sci. J. Hainan Univ..

[19] Shapawi R., Ebi I., Yong A.S.K., Ng W.K. (2014). Optimizing the growth performance of brown-marbled grouper, *Epinephelus fuscoguttatus* (Forskal), by varying the proportion of dietary protein and lipid levels. Anim. Feed Sci. Technol..

[20] Liang H.F., Huang D.K., Wu Y.H., Wang C.Q., Zhong W.J. (2013). Effects of temperature and salinity on survival and food intake of grouper hybrid (*Epinephelus lanceolatus* ♂ × *E. fuscoguttatus* ♀). J. Guangdong Ocean Univ..

[21] Zhou T., Gui L., Liu M., Li W., Hu P., Duarte D.F.C., Niu H., Chen L. (2019). Transcriptomic responses to low temperature stress in the *Nile tilapia*, *Oreochromis niloticus*. Fish Shellfish Immunol..

[22] Jiang S., Wu X., Li W., Wu M., Luo Y., Lu S., Lin H. (2015). Effects of dietary protein and lipid levels on growth, feed utilization, body and plasma biochemical compositions of hybrid grouper (*Epinephelus lanceolatus* ♂ × *Epinephelus fuscoguttatus* ♀) juveniles. Aquaculture.

[23] Xu D., You Q., Chi C., Luo S., Song H., Lou B., Takeuchi Y. (2018). Transcriptional response to low temperature in the yellow drum (*Nibea albiflora*) and identification of genes related to cold stress. Comp. Biochem. Physiol. Part D Genom. Proteom..

[24] Song S.G., Chi S.Y., Tan B.P., Liang G.L., Lu B.Q., Dong X.H., Yang Q.H., Liu H.Y., Zhang S. (2018). Effects of fishmeal replacement by Tenebrio molitor meal on growth performance, antioxidant enzyme activities and disease resistance of the juvenile pearl gentian grouper (*Epinephelus lanceolatus* ♂ × *Epinephelus fuscoguttatus* ♀). Aquac. Res..

[25] Deng Y., Zhang Y., Chen H., Xu L., Wang Q., Feng J. (2020). Gut–liver immune response and gut microbiota profiling reveal the pathogenic mechanisms of vibrio harveyi in pearl gentian grouper (*Epinephelus lanceolatus* ♂ × *E. fuscoguttatus* ♀). Front. Immunol..

[26] Xing J., Zhang Z., Sheng X., Tang X., Chi H., Zhan W. (2020). Identification and characterization of a new strain of nervous necrosis virus isolated from pearl gentian grouper (*Epinephelus lanceolatus* × *Epinephelus fuscoguttatus*) in China. Aquaculture.

[27] Zhu K., Zhang D., Wei J., Huang G., Guo Y., Jiang S. (2016). The complete mitochondrial genome of the hybrid grouper *Epinephelus fuscoguttatus* (♀)×*Epinephelus lanceolatus* (♂). Mitochondrial DNA Part A DNA Mapp. Seq. Anal..

[28] Gordon S.P., Tseng E., Salamov A., Zhang J., Meng X., Zhao Z., Kang D., Underwood J., Grigoriev I.V., Figueroa M. (2015). Widespread polycistronic transcripts in fungi revealed by single-molecule mRNA sequencing. PLoS ONE.

[29] Salmela L., Rivals E. (2014). LoRDEC: Accurate and efficient long read error correction. Bioinformatics.

[30] Li W., Godzik A. (2006). Cd-hit: A fast program for clustering and comparing large sets of protein or nucleotide sequences. Bioinformatics.

[31] Buchfink B., Xie C., Huson D.H. (2014). Fast and sensitive protein alignment using DIAMOND. Nat. Methods.

[32] Langmead B., Salzberg S.L. (2012). Fast gapped-read alignment with Bowtie 2. Nat. Methods.

[33] Li B., Dewey C.N. (2011). RSEM: Accurate transcript quantification from RNA-Seq data with or without a reference genome. BMC Bioinform..

[34] Love M.I., Huber W., Anders S. (2014). Moderated estimation of fold change and dispersion for RNA-seq data with DESeq2. Genome Biol..

[35] Shinkawa T., Taoka M., Yamauchi Y., Ichimura T., Kaji H., Takahashi N., Isobe T. (2005). STEM: A software tool for large-scale proteomic data analyses. J. Proteome Res..

[36] Wang R., Du X., Zhi Y. (2019). Screening of critical genes involved in metastasis and prognosis of high-grade serous ovarian cancer by gene expression profile data. J. Comput. Biol..

[37] Yu L., Wei M., Li F. (2020). Longitudinal analysis of gene expression changes during cervical carcinogenesis reveals potential therapeutic targets. Evol. Bioinform..

[38] Langfelder P., Horvath S. (2008). WGCNA: An R package for weighted correlation network analysis. BMC Bioinform..

[39] Fu M.C., Li H., Chen Y.Z., Liu Z.J., Liu R.Z., Wang L.G. (2020). Identification of co-expressed modules of cotton genes responding to *Verticillium dahliae* infection by WGCNA. Acta Agron. Sin..

[40] Shannon P., Markiel A., Ozier O., Baliga N.S., Wang J.T., Ramage D., Amin N., Schwikowski B., Ideker T. (2003). Cytoscape: A Software environment for integrated models of biomolecular interaction networks. Genome Res..

[41] Livak K.J., Schmittgen T.D. (2001). Analysis of Relative Gene Expression Data Using Real-Time Quantitative PCR and the 2−ΔΔCT Method. Methods.

[42] Qian B., Xue L. (2016). Liver transcriptome sequencing and de novo annotation of the large yellow croaker (*Larimichthy crocea*) under heat and cold stress. Mar. Genom..

[43] Zhou P., Li Q., Liu G., Xu N., Yang Y., Zeng W., Chen A., Wang S. (2019). Integrated analysis of transcriptomic and metabolomic data reveals critical metabolic pathways involved in polyphenol biosynthesis in *Nicotiana tabacum* under chilling stress. Funct. Plant Biol..

[44] Wang J.Y. (2002). Biochemistry.

[45] Latorre-Muro P., Baeza J., Armstrong E.A., Hurtado-Guerrero R., Corzana F., Wu L.E., Sinclair D.A., López-Buesa P., Carrodeguas J.A., Denu J.M. (2018). Dynamic acetylation of phosphoenolpyruvate carboxykinase toggles enzyme activity between gluconeogenic and anaplerotic reactions. Mol. Cell.

[46] Lamont B.J., Visinoni S., Fam B.C., Kebede M., Weinrich B., Papapostolou S., Massinet H., Proietto J., Favaloro J., Andrikopoulos S. (2006). Expression of human fructose-1,6-bisphosphatase in the liver of transgenic mice results in increased glycerol gluconeogenesis. Endocrinology.

[47] Matern D., Seydewitz H.H., Bali D., Lang C., Chen Y.T. (2002). Glycogen storage disease type I: Diagnosis and phenotype/genotype correlation. Eur. J. Pediatr. Suppl..

[48] Jobin M.L., Bonnafous P., Temsamani H., Dole F., Grélard A., Dufourc E.J., Alves I.D. (2013). The enhanced membrane interaction and perturbation of a cell penetrating peptide in the presence of anionic lipids: Toward an understanding of its selectivity for cancer cells. Biochim. Biophys. Acta.

[49] Kepinska M., Gdula-Argasinska J., Dabrowski Z., Szarek M., Pilch W., Kreska-Korus A., Szygula Z. (2017). Fatty acids composition in erythrocyte membranes of athletes after one and after a series of whole body cryostimulation sessions. Cryobiology.

[50] Cheng C.H., Ye C.X., Guo Z.X., Wang A.L. (2017). Immune and physiological responses of pufferfish (*Takifugu obscurus*) under cold stress. Fish Shellfish Immunol..

[51] Sun Z., Tan X., Liu Q., Ye H., Zou C., Xu M., Zhang Y., Ye C. (2019). Physiological, immune responses and liver lipid metabolism of orange-spotted grouper (*Epinephelus coioides*) under cold stress. Aquaculture.

[52] Zhai Z.H. (2011). Cell Biology.

[53] Sun Z., Tan X., Xu M., Liu Q., Ye H., Zou C., Ye C. (2019). Liver transcriptome analysis and de novo annotation of the orange-spotted groupers (*Epinephelus coioides*) under cold stress. Comp. Biochem. Physiol. Part D Genom. Proteom..

[54] Long Y., Song G., Yan J., He X., Li Q., Cui Z. (2013). Transcriptomic characterization of cold acclimation in larval zebrafish. BMC Genom..

[55] Cantó C., Auwerx J. (2010). AMP-activated protein kinase and its downstream transcriptional pathways. Cell. Mol. Life Sci..

[56] Gobin S., Thuillier L., Jogl G., Faye A., Tong L., Chi M., Bonnefont J.P., Girard J., Prip-Buus C. (2003). Functional and structural basis of carnitine palmitoyltransferase 1A deficiency. J. Biol. Chem..

[57] Cuccioloni M., Mozzicafreddo M., Spina M., Tran C.N., Falconi M., Eleuteri A.M., Angeletti M. (2011). Epigallocatechin-3-gallate potently inhibits the in vitro activity of hydroxy-3-methyl-glutaryl-CoA reductase. J. Lipid Res..

[58] Farhan M., Wang H., Gaur U., Little P.J., Xu J., Zheng W. (2017). FOXO signaling pathways as therapeutic targets in cancer. Int. J. Biol. Sci..

[59] Amato R., D’Antona L., Porciatti G., Agosti V., Menniti M., Rinaldo C., Costa N., Bellacchio E., Mattarocci S., Fuiano G. (2009). Sgk1 activates MDM2-dependent p53 degradation and affects cell proliferation, survival, and differentiation. J. Mol. Med..

[60] Hahn W.H., Suh J.S., Cho S.H., Cho B.S., Kim S. (2010). Do Polymorphisms of signal transducers and activators of transcription 1 and 4 (STAT1 and STAT4) contribute to progression of childhood IgA nephropathy. Cytokine.

[61] Gremer L., Merbitz-Zahradnik T., Dvorsky R., Cirstea I.C., Kratz C.P., Zenker M., Wittinghofer A., Ahmadian M.R. (2011). Germline KRAS mutations cause aberrant biochemical and physical properties leading to developmental disorders. Hum. Mutat..

[62] McGrath D.A., Fifield B., Marceau A.H., Tripathi S., Porter L.A., Rubin S.M. (2017). Structural basis of divergent cyclin-dependent kinase activation by Spy1/ RINGO proteins. EMBO J..

[63] Takekawa M., Saito H. (1998). A family of stress-inducible GADD45-like proteins mediate activation of the stress-responsive MTK1/MEKK4 MAPKKK. Cell.

[64] Hu P., Liu M., Liu Y., Wang J., Zhang D., Niu H., Chen L. (2016). Transcriptome comparison reveals a genetic network regulating the lower temperature limit in fish. Sci. Rep..

[65] Liebermann D.A., Hoffman B. (2007). Gadd45 in the response of hematopoietic cells to genotoxic stress. Blood Cells Mol. Dis..

[66] Li C., Gu Y., Tang S., Fang H., Jiang G., Jiang Z. (2011). Effects of acute low temperature stress on the endocrine reactions of the Qinghai toad-headed lizard. Curr. Zool..

[67] Berglund E.D., Kang L., Lee-Young R.S., Hasenour C.M., Lustig D.G., Lynes S.E., Donahue E.P., Swift L.L., Charron M.J., Wasserman D.H. (2010). Glucagon and lipid interactions in the regulation of hepatic AMPK signaling and expression of PPARα and FGF21 transcripts in vivo. Am. J. Physiol. Endocrinol. Metab..

[68] Talbert M.E., Langefeld C.D., Ziegler J., Mychaleckyj J.C., Haffner S.M., Norris J.M., Bowden D.W. (2009). Polymorphisms near SOCS3 are associated with obesity and glucose homeostasis traits in Hispanic Americans from the Insulin Resistance Atherosclerosis Family Study. Hum. Genet..

[69] Baldi P., Valè G., Mazzucotelli E., Govoni C., Faccioli P., Stanca A.M., Cattivelli L. (2001). The transcripts of several components of the protein synthesis machinery are cold-regulated in a chloroplast-dependent manner in barley and wheat. J. Plant Physiol..

[70] Haque M.S., Haque M.A., Roy S.K., Khan M.M.H., Hossain M.M. (2014). Regulatory mechanism on enhancing protein synthesis in skeletal muscles of cold exposed fresh water fish (*Channa punctata*). J. Saudi Soc. Agric. Sci..

